# Animal Models for Neuroinflammation and Potential Treatment Methods

**DOI:** 10.3389/fneur.2022.890217

**Published:** 2022-06-27

**Authors:** Yasuhisa Tamura, Masanori Yamato, Yosky Kataoka

**Affiliations:** ^1^Laboratory for Cellular Function Imaging, RIKEN Center for Biosystems Dynamics Research, Kobe, Japan; ^2^Multi-Modal Microstructure Analysis Unit, RIKEN-JEOL Collaboration Center, RIKEN, Kobe, Japan

**Keywords:** myalgic encephalomyelitis/chronic fatigue syndrome, lipopolysaccharide, polyinosinic-polycytidylic acid, inflammation, cytokine

## Abstract

Myalgic encephalomyelitis/chronic fatigue syndrome (ME/CFS) is a debilitating chronic disease of unknown etiology and without effective treatment options. The onset of ME/CFS is often associated with neuroinflammation following bacterial or viral infection. A positron emission tomography imaging study revealed that the degree of neuroinflammation was correlated with the severity of several symptoms in patients with ME/CFS. In animal studies, lipopolysaccharide- and polyinosinic-polycytidylic acid-induced models are thought to mimic the pathological features of ME/CFS and provoke neuroinflammation, characterized by increased levels of proinflammatory cytokines and activation of microglia. In this review, we described the anti-inflammatory effects of three compounds on neuroinflammatory responses utilizing animal models. The findings of the included studies suggest that anti-inflammatory substances may be used as effective therapies to ameliorate disease symptoms in patients with ME/CFS.

## Introduction

ME/CFS is a clinically complex and chronic condition characterized by unexplained fatigue and post-exertional malaise with symptoms including pain, sleep disturbance, and cognitive, neuroendocrine, gastrointestinal, and immune dysfunction (1, 2). Viral or bacterial infection is closely related to the pathogenesis of ME/CFS. In fact, previous studies have reported that infection with Epstein-Barr virus, human herpesvirus 6, cytomegalovirus, enterovirus (3–7), as well as bacteria (8–10), is involved in the maladaptive progression of ME/CFS. Viral or bacterial infections cause inflammatory responses in the brain (neuroinflammation) as well as in peripheral tissues. Imaging studies done in patients with ME/CFS have shown that the degree of neuroinflammation is correlated with the severity of several symptoms including pain, depression, and cognitive impairment (11).

Neuroinflammation is involved in the onset and/or progression of several neurodegenerative and neuropsychiatric disorders (12–16) and is facilitated by microglia activation and elevated expression of proinflammatory cytokines, including interleukin-1β (IL-1β), IL-6, and tumor necrosis factor-α (TNF-α) (17–20). Studies using animal models have shown that systemic and central administration of lipopolysaccharide (LPS) and polyinosinic-polycytidylic acid (poly I:C) can induce neuroinflammation through upregulation of proinflammatory cytokines and glial activation (21–27). These models can be used for evaluating the anti-inflammatory potential of therapeutics, healthy foods, and nutraceutical products. This review describes the anti-inflammatory effects of three different types of compounds: IL-1 receptor antagonist (IL-1ra), minocycline, and 6- (methyl sulfinyl) hexyl isothiocyanate (6-MSITC).

## Role of Neuroinflammation in Myalgic Encephalomyelitis/Chronic Fatigue Syndrome (ME/CFS)

Although 0.5–1.5% of people globally suffer from ME/CFS, identification of abnormal factors in ME/CFS is difficult *via* general and conventional medical examination. Such obstacles delay diagnosis in many patients and could take several months, or years, to obtain a definite ME/CFS diagnosis. Furthermore, effective treatments have yet to be established (28).

To date, the pathophysiological mechanisms of ME/CFS have been assumed to be facilitated by viral infections, immunological abnormality, oxidative stress, and impaired energy metabolism with reduced production of mitochondrial adenosine-5′-triphosphate (29–35). Morphological changes and abnormal functionality in the brain have also been reported, particularly in imaging and psychiatric studies (36). Specifically, a MRI study revealed prefrontal cortical atrophy in patients with ME/CFS (37). Further, positron emission tomography (PET) imaging studies have suggested a reduction in the biosynthesis of neurotransmitters through estimation of acetyl-L-carnitine and serotonin transporter densities in the brains of individuals with ME/CFS (38, 39).

In addition to the observed pathological phenomena, we hypothesized that neuroinflammation is involved in the pathophysiology of ME/CFS, on the basis of experimental observations in animals showing fatigue- or depression-like behavior after proinflammatory cytokine production by activated microglia in the brain (40), although there has been no direct evidence of neuroinflammation in ME/CFS. Nakatomi et al., first demonstrated that neuroinflammation was widely induced in patients with ME/CFS by using PET with PK11195, a PET tracer for activated microglia (11). Furthermore, neuroinflammation was associated with the severity of neuropsychological symptoms including the following: severity in the amygdala was correlated with the cognitive impairment score; severity in the hippocampus was related to the depression score; and severity in the thalamus was related to the pain score. Detection of neuroinflammation in patients with ME/CFS may be essential for an objective diagnosis and deciding the medical treatment strategy for ME/CFS, as well as for understanding the underlying pathophysiological mechanism. In the ongoing coronavirus disease (COVID-19) pandemic, individuals are suffering from long-lasting symptoms including “brain fog,” which is deemed long COVID or post-COVID-19 syndrome. It has been suggested that this pandemic has increased the prevalence of ME/CFS with prolonged or intermittent neuroinflammation (41, 42).

## Animal Models for Neuroinflammation

**Figure 1:**
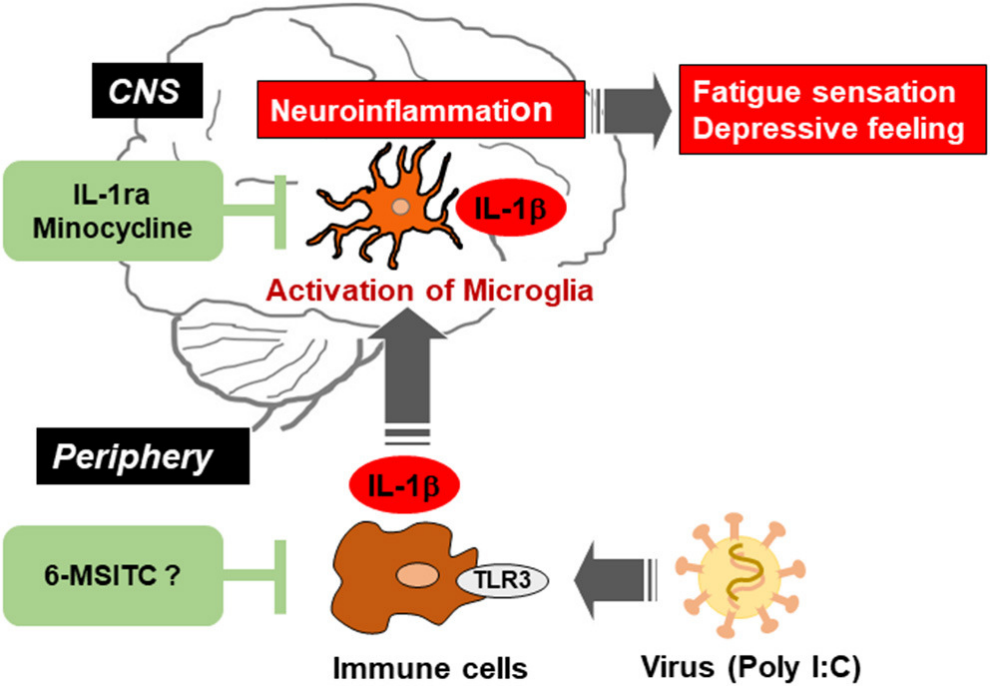
Schematic illustration indicating the viral infection-induced neuroinflammation in the brain *via* activation of microglia, and the possible therapeutic points by IL-1ra, minocycline, and 6-MSITC. Virus activates toll-like receptor 3 (TLR3) in immune cells and triggers the production of proinflammatory cytokines including IL-1β. Such cytokines in the periphery induce activation of microglia and production of IL-1β in the central nervous system (CNS). Neuroinflammation could be alleviated by suppressing immune response in peripheral immune cells and/or microglia in CNS.

Neuroinflammation is defined as an inflammatory response in the central nervous system, including the brain and spinal cord, and plays a crucial role in the pathogenesis of several diseases including Alzheimer's disease, Parkinson's disease, depression, and ME/CFS. In experimental studies, toll-like receptor (TLR) ligand-induced inflammation is used as an animal model of neuroinflammation (Figure 1). Similarly, LPS and poly I:C models are thought to mimic the pathological relevance of ME/CFS (43, 44), potentially through TLR signaling. LPS is a TLR4 ligand and main component of the outer membrane of Gram-negative bacteria. In rodents, a peripheral LPS challenge induced expression of proinflammatory cytokine expression at the gene and protein level, and evoked activation of microglia/macrophages in the brain as well as in peripheral tissues (19, 45). In general, LPS injected systemically does not pass through the blood-brain barrier (BBB). Therefore, signal transduction in the periphery may facilitate the induction of inflammatory reactions in the brain (46, 47). Central (intracerebroventricular or intraparenchymal) administration of LPS can directly and acutely induce inflammatory brain reactions (48). Indeed, a peripheral LPS challenge elevated expression of proinflammatory cytokines (IL-1β, IL-6, and TNF-α) in the brain at 2 h, as well as increasing plasma IL-6 levels (48). This indicated that central LPS-induced neuroinflammation may be linked to the induction of peripheral inflammatory responses.

On the other hand, poly I:C, a synthetic double-stranded RNA, can be used to mimic viral infections through TLR3 (43, 49, 50). As with LPS, systemic administration of poly I:C promoted increased expression of proinflammatory cytokines (IL-1β, IL-6, and TNF-α) in the brain and serum (21, 22, 51). Recently, it has been shown that a central poly I:C challenge provoked upregulation of proinflammatory cytokines (IL-1β, IL-6, and TNF-α) in the brain, with maximal gene expression peaking at 4 h (25). These findings suggest that peripheral and central administration of LPS and poly I:C can induce inflammatory responses in the brain and can be used as neuroinflammatory animal models for the screening of anti-inflammatory drugs and foods.

## Treatments for Neuroinflammation

Herein we describe the anti-inflammatory effects of three compounds, IL-1ra, minocycline, and 6-MSITC, on LPS- or poly I:C-induced neuroinflammation.

IL-1ra is a member of the IL-1 family and is known as an endogenous competitive antagonist for IL-1 receptors, that is, IL-1ra counteracts the action of IL-1β (52). We previously investigated that poly I:C-induced decrease in locomotor activity was completely blocked by intracerebroventricular (i.c.v) infusion of recombinant IL-1ra (40). Also, we demonstrated that the recovery from a decrease in spontaneous activity during poly I:C-induced neuroinflammation was significantly delayed by i.c.v. infusion of a neutralizing antibody for endogenous IL-1ra. These results indicate that endogenous IL-1ra in the brain has an important role in the prevention of prolonged inflammation (Figure 1). Recent studies have shed light on neuroinflammation as an essential precipitating event in nervous system diseases including ME/CFS (11, 53, 54). Therefore, a balance between the production of IL-1β and its endogenous antagonist could regulate neuroinflammation and decrease locomotor activity. These reports enhance our understanding of how neuroinflammation could shift from an acute to a chronic state.

Minocycline is known as a second-generation and semi-synthetic tetracycline antibiotic. Minocycline is quickly absorbed into the body, penetrates the BBB, and affects many biological actions (differing from its antibiotic action) both *in vivo* and *in vitro*, including the following: attenuation of BBB breakdown by inhibiting the production of matrix metalloproteinase-9; functional improvement after traumatic brain injury *via* suppression of aquaporin-4 production; relieve white matter injury in the neonatal rat brain by suppression of IL-1β and TNF-α production; neuroprotection from ischemic brain damage; alleviation of LPS-induced depressive-like behavior; and suppression of NOx production in cultured microglia under hypoxia (55, 56). In our study, we demonstrated that intraperitoneal pretreatment with minocycline (20 mg/kg/day, 3 consecutive days) attenuated poly I:C-induced IL-1β mRNA expression in rat brain, transient fever, and decrease in locomotor activity (57). Further, it was also reported that intrathecal pretreatment with minocycline attenuated chronic stress-induced muscular hyperalgesia and mechanical allodynia by suppression of spinal cord microglial activation in rat model for ME/CFS (58). These observations suggest that minocycline could be a new drug for improving some deficits seen in neurological disorders (Figure 1).

Although the mechanisms underlying minocycline's anti-inflammatory effect on neuroinflammation are not well-understood, once severe neuroinflammation occurs, suppression might prove to be difficult. Indeed, we could not demonstrate suppression of neuroinflammation by minocycline after poly I:C-injection without pretreatment (57). A better understanding of minocycline's role in neuroinflammation is required to maximize its therapeutic potential. Overall, control of neuroinflammation will alleviate fatigue and chronic pain, and contribute to preventing the progression of neurological disorders, including ME/CFS.

Finally, 6-MSITC derived from Wasabi (*Wasabia japonica*) is a naturally occurring compound that has several biological functions, including anti-inflammatory, antitumor, and anticoagulant activities. *In vitro* studies have shown that 6-MSITC treatment inhibited activation of murine macrophage cells following the application of LPS (59), and attenuated TNF-induced upregulation of IL-6 in human umbilical vein endothelial cells (60). In addition, 6-MSITC alleviated several inflammatory responses in a murine model of inflammatory bowel disease, known as chronic inflammatory disorders of the gastrointestinal tract (61). However, the anti-inflammatory effects of 6-MSITC on LPS- or poly I:C-induced neuroinflammation have not been characterized. In our studies, long-term use of 6-MSITC relieved neuroinflammatory responses following a peripheral injection of poly I:C (Figure 1), but did not show anti-inflammatory effects on neuroinflammation following a central LPS challenge (62). The observed discrepancy is unclear but warrants further investigation. Still, these findings suggest that 6-MSITC may ameliorate the neuropsychological symptoms of ME/CFS with viral infections.

Recently, the gut microbiota has been reported to be closely associated with the ME/CFS pathophysiology, including neuroinflammation and cognitive symptoms (63, 64). Long-term complications of long COVID or post-COVID-19 syndrome have also been attributed to the dysregulation of the gut microbiota (65, 66). Minocycline may suppress neuroinflammation by affecting the gut microbiome and intestinal permeability, as shown in a rat model of Gulf War illness (67). Peripheral IL-1ra also plays a crucial role in the regulation of the gut microbiota (68), although we employed the direct i.c.v. infusion of recombinant IL-1ra for the prevention of prolonged neuroinflammation in our animal study introduced in this review (40). Therefore, these findings suggest that the gut microbiota should be taken into consideration in animal models for ME/CFS. It is expected that effective food nutrients and/or ingredients as well as medicinal drugs will be discovered for the treatment of neuroinflammation in ME/CFS and long COVID.

## Conclusions

ME/CFS is a complex multi-system illness without diagnostic markers or efficacious therapy options. The pathogenesis of ME/CFS is linked with viral or bacterial infection-induced neuroinflammation. In animal studies, LPS and poly I:C models can be used as infection-induced neuroinflammation and can promote increased expression of proinflammatory cytokines and microglia activation. Neuroinflammation is thought to be mitigated by suppressing immune response in the central nervous system as well as in the periphery. Herein, we reviewed the anti-inflammatory effects of three compounds using animal models and highlight the potential of these anti-inflammatory drugs to alleviate symptoms in patients with ME/CFS.

## Author Contributions

YT and YK designed the figures. All authors wrote and corrected the manuscript. All authors have agreed to the submitted version of manuscript.

## Funding

This work was supported in part by JSPS KAKENHI Grant Numbers 16K10232 and 20K06883 to YT, JP13351328 and JP18963739 to YK, and Cross-ministerial Strategic Innovation Promotion (SIP) to YK.

## Conflict of Interest

The authors declare that the research was conducted in the absence of any commercial or financial relationships that could be construed as a potential conflict of interest.

## Publisher's Note

All claims expressed in this article are solely those of the authors and do not necessarily represent those of their affiliated organizations, or those of the publisher, the editors and the reviewers. Any product that may be evaluated in this article, or claim that may be made by its manufacturer, is not guaranteed or endorsed by the publisher.

## Abbreviations

IL-1β: Interleukin-1β
IL-6: Interleukin-6
ME/CFS: Myalgic encephalomyelitis/chronic fatigue syndrome
TNF-α: Tumor necrosis factor-α
LPS: Lipopolysaccharide
Poly I:C: Polyinosinic-polycytidylic acid
IL-1ra: IL-1 receptor antagonist
6-MSITC: 6-(methyl sulfinyl) hexyl isothiocyanate
TLR: Toll-like receptor
BBB: Blood-brain barrier
i.c.v: Intracerebroventricular.
